# Fish alter locomotor and feeding kinematics to capture aerial prey

**DOI:** 10.1098/rspb.2025.1308

**Published:** 2025-11-26

**Authors:** Erik G. Axlid, Christian Lowe, Ethan S. Wang, Timothy E. Higham

**Affiliations:** ^1^ Department of Evolution, Ecology and Organismal Biology, University of California, Riverside, Riverside, CA 92521, USA

**Keywords:** fish feeding, aerial prey capture, jumping, predator–prey interactions, largemouth bass

## Abstract

Although most fishes feed exclusively in water, some are capable of leaping into the air to capture flying, suspended or perched prey. Whereas some species specialize in aerial prey capture, others will only opportunistically feed aerially and lack any known morphological specializations that enhance their ability to do so. A key outstanding question is how non-specialist species overcome the challenges associated with aerial feeding, including the visual disruption at the air-water interface and the drastic differences in fluid density. Using largemouth bass (*Micropterus salmoides*), we addressed whether there are kinematic differences between aerial and aquatic feeding, and how generalist fishes modulate their kinematics to catch aerial prey at different heights. We quantified the three-dimensional kinematics of bass capturing mealworms aquatically and at a range of heights above the surface. Aerial feeding involved larger, faster cranial movements than aquatic prey capture. In addition, feeding events aimed at prey suspended higher were characterized by higher peak velocities and accelerations, as well as more propulsive tail strokes. Our results reveal how generalist fishes can feed in drastically disparate physical environments and will help us understand the evolution of aerial prey capture by examining exaptation to aerial feeding in primarily aquatic feeders.

## 1. Background

Fishes capture a diverse range of prey types, from zooplankton and plant matter to rodents and anurans, using a wide variety of prey capture strategies [1–4]. Three feeding modes have been widely studied: suction, ram and manipulation [4,5]. During suction feeding, which is used to some extent by nearly all teleosts [4], fish position the aperture of their mouth close to the prey and rapidly expand their buccal and opercular cavities, creating a sudden drop in pressure that draws water into the mouth [6]. In a successful feeding attempt, the resulting pressure gradient and flow of water exert force on the prey, drawing it into the predator’s buccal cavity [7,8]. Ram feeding does not require any suction-induced movement of the prey, and ram feeders instead accelerate their jaws and body to overtake and engulf their target, while manipulation (also termed ‘biting’) requires predators to use their oral jaws to remove pieces from a larger prey item or smaller organisms from their substrate [9–11].

Some unique modes of fish feeding, however, such as those that occur during emersion, do not fit into any of these categories and exist outside of the traditional framework. Fishes that feed during emersion can either capture prey by grasping and ingesting them from the substrate with their oral jaws [12,13], or jump above the surface to capture flying or suspended insects and small vertebrates during what is termed ‘aerial feeding’ [14]. Aerial feeding is employed by a diverse range of species [15–19], but studies have largely focused on the smallscale archerfish (*Toxotes microlepis*) [20] and silver arowana (*Osteoglossum bicirrhosum*) [14].

Aerial feeding presents at least two major challenges, which may explain why relatively few taxa feed terrestrially or aerially [12]. First, suction feeding is practically impossible outside of aquatic environments [21]. The low density of air would require fishes to expand their buccal cavities 28 times faster than they do in water to produce flows with the same kinetic energy, which is thought to be an order of magnitude greater than muscle physiology allows [22,23]. Second, refraction shifts the apparent location of the prey higher when viewed from below the surface, an effect that becomes more pronounced at more acute angles relative to the surface according to Snell’s law [24]. Though archerfish are known to adjust their aim to compensate for this distortion [24–26], it is unknown whether other fishes can make similar corrections.

The kinematics underlying aerial feeding have been described in only two taxa, both of which regularly feed aerially [27,28] and have specialized mouth, jaw or sensory morphologies not common among teleosts [25,29–31]. The silver arowana (*O. bicirrhosum*), an elongate South American fish that can capture birds perched on overhanging branches [27], uses S-starts to produce the sudden thrust required to jump. During aerial feeding, *O. bicirrhosum* generally displays larger and faster jaw movements than those used in aquatic strikes, due either to specific requirements of feeding aerially versus aquatically or to the differences in density and viscosity between air and water [14,32]. Archerfish (*T. microlepis*) feeding aerially increase the number of propulsive caudal strokes, as well as the peak acceleration and velocity, of their jump approach when the prey is positioned higher above the surface [20]. Additionally, although their jumps serve different purposes, migrating salmonids jump higher when they initiate jumps from deeper pools, likely because deeper waters add to the distance over which they can accelerate [33–35]. Trinidadian guppies (*Poecilia reticulata*), which jump spontaneously, similarly jump higher when they swim backwards farther in the preparatory phase prior to jumping [36], a manoeuvre that usually increases their available acceleration distance.

Almost nothing is known about how non-specialized species that primarily feed aquatically can overcome the physical challenges associated with aerial feeding, despite the prevalence of this feeding mode across diverse taxonomic groups [15,16,18]. Largemouth bass have been a model for studying fish feeding for decades [37–41] and occasionally feed aerially [42], though they have a generalist body form without any known morphological specializations previously linked to this ability. These factors make *M. salmoides* an ideal study system for examining aerial feeding in non-specialists. Understanding how some generalist species are able to opportunistically feed aerially could help identify exaptation to aerial feeding in primarily aquatic feeders, thereby contributing to our understanding of the evolution of biomechanical diversity in fishes.

In the present study, we addressed whether there are kinematic differences between aerial and aquatic feeding, as well as how the kinematics of aerial feeding are modulated with differences in prey height. We predicted that aerial feeding in largemouth bass would involve larger and faster movements of the jaw and associated elements than aquatic feeding, and that aerial feeding would require kinematic shifts toward a feeding strategy not reliant on suction due to the physical limitations of a low-density medium [14,21]. In addition, we predicted that jump height, the associated take-off velocity, and acceleration, are tuned to prey height and achieved via more propulsive tail strokes and deeper attack initiation depths [20]. This study will yield valuable information about the morphological requirements for feeding aerially and deepen our understanding of how organisms with the ability to operate under a wide array of physical conditions can expand their niches to gain competitive advantages.

## 2. Material and methods

### (a) Animal acquisition and husbandry

Juvenile largemouth bass were acquired from Kent SeaTech (Mecca, CA, USA) approximately 3 months prior to the start of experiments. The fish were housed individually in 10 gallon (approximately 37.8 l) aquaria filled with treated tap water, fed slow-sinking cichlid pellets (Aqueon Products, Franklin, WI, USA) every other day, and exposed to natural lighting conditions. Water temperatures remained steady at approximately 20°C. At the time of all experiments, the mean standard length was 17.01 ± 1.14 cm (mean ± s.d.). All procedures involving live animals were approved by the University of California, Riverside’s Institutional Animal Care and Use Committee (IACUC) protocol #117.

### (b) Videography

**Figure 1.**
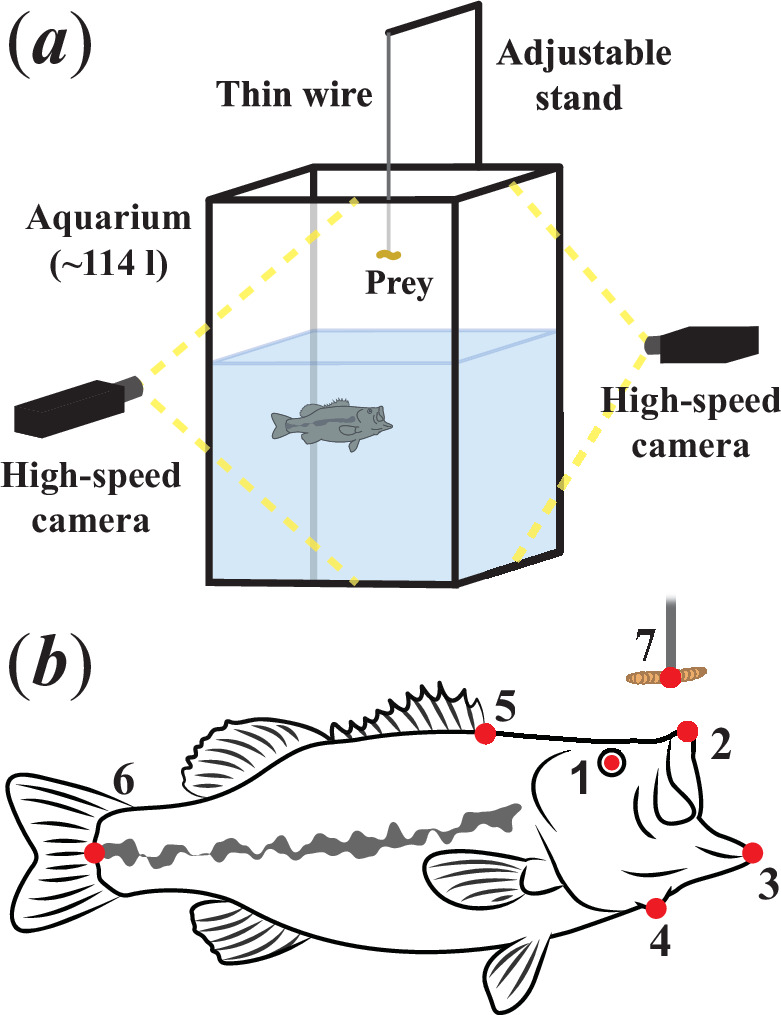
(*a*) Schematic diagram of the experimental set-up showing the aquarium, cameras and the apparatus used to suspend prey. (*b*) Morphological landmarks tracked during prey capture. See main text for definitions.

We obtained stereoscopic high-speed video of 14 largemouth bass, each feeding both aerially and aquatically on mealworms, at 500 frames per second (fps). The stereoscopic view was accomplished using two Edgertronic SC1 cameras separated by an angle of approximately 90° relative to the prey. During the aerial feeding trials, the cameras were positioned level with the surface and pointed perpendicularly to the side of the aquarium to minimize distortion (figure 1). Aerial feeding trials were conducted in a 30 gallon (approximately 114 l) aquarium measuring approximately 60 × 30 × 60 cm (length × width × height) and filled with treated tap water to a depth of 40 cm, while aquatic feeding trials were conducted in a full 20 gallon (approximately 71 l) tank measuring approximately 77 × 33 × 33 cm. Aquariums with these dimensions were chosen to allow for maximal freedom of movement along the primary axes of motion during both aerial (vertical) and aquatic (horizontal) feeding. Given the location where the prey was positioned during the aquatic trials, the starting distance between the predator and prey was almost identical in the two tanks. The area within which the largemouth bass fed aquatically was calibrated using a custom calibration object with 18 points occupying regions that covered the attack volume. For aerial feeding, six additional points were added to the object near the air/water interface to minimize the effects of optical distortions across the surface.

### (c) Experimental design

Three days prior to planned aerial feeding trials, individual fish were transferred by dip net to the experimental aquarium for acclimation. The next day, they were fed five mealworms at increasing heights to verify that acclimation had occurred and that stress caused by handling would not prevent feeding. If the fish did not consistently feed aerially at a prey height of at least 5 cm, this process was repeated the next day until this was achieved. If acclimated, the fish were fed pellets and subsequently starved for approximately 48 h.

After this starvation period, aerial feeding trials began. Feeding events were elicited by suspending a mealworm, pierced on a thin wire, above the surface. The mealworms were sometimes nudged to draw the attention of the fish but were stationary during feeding attempts.

Aerial feeding events were recorded at three prey heights, measured vertically from the surface using a ruler and later confirmed through video landmark analysis. Each fish was first prompted to feed on prey suspended at 10 cm. If the mealworm was successfully captured at this height, the fish was given a maximum prey capture height test, and lastly, a mealworm suspended at 5 cm. If the prey was not captured at its initial height of 10 cm for a period of 5 min, the 5 cm attempt was given first, before the prey was returned to a height of 10 cm. If the mealworm was successfully captured during this second period, the maximum prey capture height test was administered starting at this height, and if not, the maximum prey capture height test began at 5 cm.

As a result of this procedure, the maximum prey capture height test always began following a successful aerial feeding event at either 5 or 10 cm above the surface. The mealworm was then raised by 2 cm, and an additional 2 cm after each successful prey capture until failure. Failure was defined as the point where no feeding attempts were made for 5 min, or after three unsuccessful feeding attempts. Upon failure, the prey was lowered by 1 cm and the incremental raise in the case of a successful attempt decreased to 1 cm (electronic supplementary material, figure S1). The test continued until failure was reached for a second time, and the maximum height at which an individual successfully captured prey was recorded.

Aquatic feeding events were captured for each fish approximately 6 weeks after their aerial feeding trials. Randomization was not possible due to unforeseen logistical challenges. During the recording of aquatic feeding, one fish at a time was acclimated to the experimental tank overnight (approximately 24 h), then positioned in one end of the aquarium away from the cameras. A mealworm was then dropped in front of the cameras, which prompted rapid swimming towards the prey followed by a strike.

### (d) Kinematic analysis

The three-dimensional coordinates of the centre of the eye, the anterior tip of the premaxilla, the anterior tip of the mandible, the ventral-most point of the buccal floor, the base of the first dorsal spine, the dorsoventral centre of the caudal peduncle and the estimated centre of mass of the mealworm (figure 1) were tracked over time using DLTdv8a (v.8.2.13) [43]. During periods in which a point was not directly visible to both cameras, the epipolar line generated by the software was used in combination with morphological inference to estimate its coordinates. A custom MATLAB (v.R2023b) script was then used to calculate relevant angles and distances. These data were smoothed using a fourth-order Butterworth filter with a cutoff frequency of 35 Hz.

Gape size was measured as the maximum distance between the anterior tip of the premaxilla and the anterior tip of the mandible. Premaxillary protrusion was the distance between the centre of the eye and the anterior tip of the premaxilla, and hyoid depression was defined as the distance between the centre of the eye and the most ventral point of the buccal floor. The distance between the anterior tip of the premaxilla and the prey was measured at two time points; at attack onset, defined as the time point at which the body began to curve for an S-start, and strike onset, defined as the time point at which jaw depression began. These two resulting distances were named the attack distance and strike distance, respectively. We also calculated strike duration (from strike onset until the mouth closed around the prey), time to onset of hyoid depression and cranial elevation (from strike initiation), time to maximum gape (from strike initiation), time to peak cranial elevation (from the onset of cranial elevation), time to peak hyoid depression (from the onset of hyoid depression), peak gape duration and duration of mouth closing (from the end of peak gape) by manually noting the timing of key kinematic events.

The custom MATLAB script was also used to quantify instantaneous velocity based on the distance travelled between frames by the point tracked at the base of the first dorsal spine. Maximum instantaneous velocity was then extracted from these data. Peak acceleration was calculated as the maximum change in velocity between two frames divided by the time between frames. These data were also smoothed using the fourth-order Butterworth filter described above. The time spent above both 50% and 75% of peak velocity for each feeding event was also recorded. The former was used in comparisons between aerial feeding at different heights, while the latter was used in comparisons between aquatic and aerial feeding events due to the relatively high and constant swimming speeds displayed during aquatic feeding.

In addition, several variables specific to aerial feeding were measured only during aerial feeding events. Peak height was defined as the maximum jump height achieved, which in turn was measured as the vertical distance between the anterior tip of the lower jaw (also the most anterior point on the fish when the mouth is closed) and the surface of the water. Prey height was the vertical distance between the approximate centre of mass of the mealworm and the surface. Strike height was the jump height at the instant of strike initiation, and overshoot was a measure of the vertical distance travelled by the fish in relation to the prey and calculated as the jump height minus prey height. The body angle of the fish at attack onset was measured as the angle between a plane representing the surface and a line connecting the points at the anterior tip of the mandible and the caudal peduncle in the frame before attack onset. The jump angle was defined as the angle between the surface and a line drawn between the base of the first dorsal spine 10 frames before and 10 frames after said spine broke the surface. Lastly, attack depth was the vertical distance between the most anterior point of the fish and the surface at the time of attack onset.

To evaluate the role of the body and tail in thrust generation during jumping, we also recorded the number of propulsive tail stroke cycles between attack initiation and prey capture. Caudal fin use during aerial feeding bouts generally began and ended with the caudal fin near the midline. Thus, one cycle was counted after the caudal fin reached its lateral maximum displacement on both the left and right sides and returned to the midline. The number of propulsive strokes was rounded to the nearest half, with a half cycle occurring when the caudal fin departed the midline, reached only its maximum displacement on one side, and returned to the centre.

### (e) Statistical analyses

All analyses were conducted using IBM SPSS Statistics 28. To reduce the dimensionality of our dataset, two principal component analyses (PCA) were used. The first was aimed at summarizing differences between aerial and aquatic feeding and included three feeding events from each fish: an aquatic event, an aerial feeding event at a prey height of 5 cm and an aerial feeding event at the maximum prey height at which a given individual successfully captured the prey, regardless of whether that maximum height was 10 cm or higher. Only variables that could be derived from both aerial and aquatic prey capture were included in this analysis.

Using one trial per condition and fish was intended to minimize any effects of learning and experience on kinematics, which would otherwise have introduced an additional independent variable, as well as to avoid pseudoreplication without the need for more complex analyses. An adequate sample size was instead obtained by maximizing the number of individuals from which data were collected. Key kinematic measurements derived from the aquatic feeding events were compared with literature values to ensure they were representative of largemouth bass feeding with maximal effort (see electronic supplementary material, table S1).

The second PCA excluded aquatic feeding and instead used three prey height categories: 5 cm, 10 cm and maximum height (if different from 10 cm). This analysis included nearly all measured variables, including those that could only be measured for aerial feeding. Due to performance differences, however, feeding events in all three categories could not be produced by every individual. Therefore, while most fish (8 of 14) were represented by three feeding events across all prey height categories in this analysis, the rest were only represented by events in two of the three categories. For both analyses, variables with loading scores with absolute values below 0.4 were excluded, in addition to peak jump height, attack distance and strike height due to their strong inherent correlations to other variables. PCA scores were compared using repeated measures ANOVA.

Repeated measures ANOVA was also used to further evaluate timing-related variables (time to peak gape, time to maximal hyoid depression, peak gape duration, duration of mouth closing and time to peak cranial elevation), strike distance and measurements of the extent of cranial movements (max gape size, peak hyoid depression). The assumption of sphericity was tested using Mauchly’s test, and the Greenhouse-Geisser correction was applied to adjust the degrees of freedom where this assumption was violated. Pairwise comparisons following all repeated measures ANOVA were made using least significant difference (LSD) testing.

To elucidate factors that contribute to aerial feeding at greater prey heights, we also used least squares linear regressions of peak velocity, peak acceleration, time spent above 50% of peak velocity and number of propulsive fin strokes against jump height, as well as overshoot, attack depth and strike height against prey height to describe the kinematic effects of varying prey height. We also used linear regressions of kinematic data from the aquatic feeding events against the time elapsed between aerial and aquatic trials to rule out any effect of time.

## 3. Results

### (a) Description of aerial feeding in *M. salmoides*

**Figure 2.**
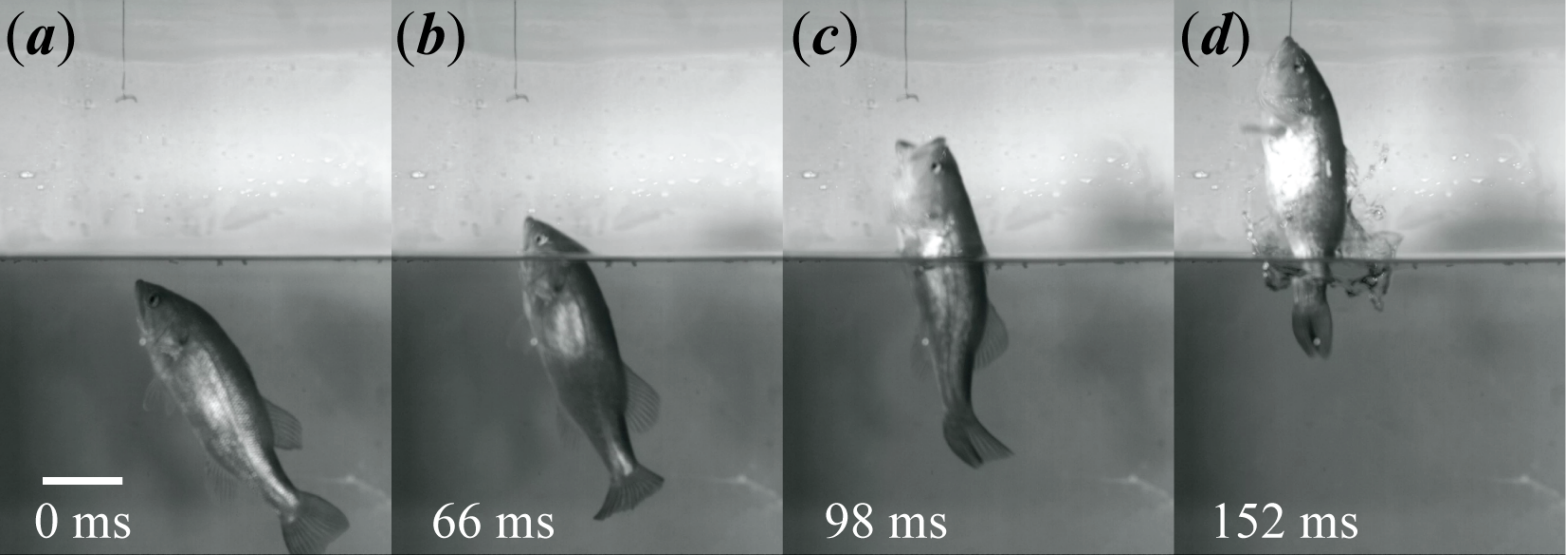
Still frames from a representative aerial feeding event (prey height 10 cm) at the time of (*a*) attack initiation, (*b*) strike initiation, (*c*) peak gape and (*d*) mouth closing. Times are shown relative to attack initiation. Scale bar = 5 cm. For a video of a maximal feeding event, see electronic supplementary material, video S1.

Prior to each aerial feeding attempt, the bass tracked the suspended mealworm by hovering with the tip of their mouth near the top of the water, occasionally (but barely) breaking the surface. The mean hovering body angle relative to the surface was 54.5 ± 6.95° (mean ± s.d.) immediately prior to the attack. Upon attack initiation, the body formed the double-bend characteristic of an S-start [44,45] and then accelerated rapidly, propelling the fish into the air. This was observed in every aerial feeding attempt. The mean jump angle was 69.4 ± 7.52°. Mouth opening generally occurred above the surface (figure 2). However, at low prey heights, the jaw occasionally began to open while the mouth was still underwater. After generating sufficient thrust through body and caudal fin undulations, the fish coasted until they engulfed the prey, closed their mouth around it and fell back into the water (see electronic supplementary material, video S1). A detailed analysis of pectoral fin motion was difficult to conduct due to disturbances in the water, but they appeared to be abducted near the apex of nearly every jump.

In 14 of the 36 analysed aerial feeding events, and most frequently at the lower prey heights, the prey passed through the oral aperture without contacting the oral jaws. In 16 events, the mealworm appeared to make contact as it passed through the mouth before closure began, and in six cases, the mouth clearly began closing before contacting the prey. The effects of contact between the predator and prey, and therefore potential tactile feedback, on strike kinematics could not be evaluated in detail.

### (b) Differences between aerial and aquatic feeding

**Figure 3.**
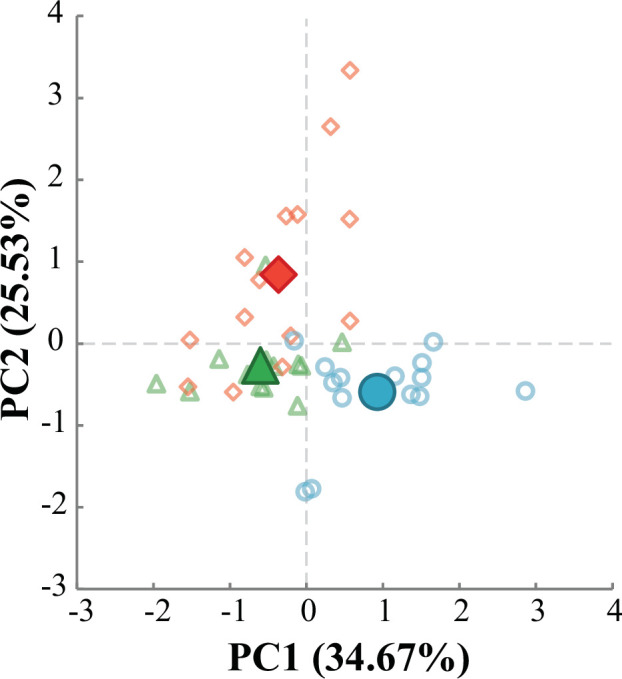
PC1 and PC2 scores for aerial and aquatic feeding events. Aquatic events are represented by open blue circles (*n* = 14), while scores for aerial feeding events at prey heights of 5 cm (*n* = 13) and at maximal height (*n* = 14) are shown as green triangles and red diamonds, respectively. Filled shapes denote mean PC scores for each prey height category. The time to completion of several jaw movements loaded positively on PC1, while peak velocity, peak acceleration and two variables indicating the extent of jaw movements loaded negatively. This component separated aquatic feeding from aerial feeding at either prey height (*F*_2,24_ = 18.811, *p* < 0.001). Time to initiation and completion of several jaw movements loaded positively on PC2, as well as peak gape duration and strike distance. Aerial feeding events at maximal prey heights scored significantly higher on PC2 than both aquatic feeding events and aerial feeding events at the lower prey height PC2 (*F*_2,24_ = 11.366, *p* < 0.001). Complete variable loadings are listed in table 1.

The PCA used to summarize differences between aerial and aquatic feeding identified two variables that together explained 60.21% of the total variance. A pair of repeated measures ANOVAs revealed that PC1 separated aquatic feeding events from aerial feeding at any prey height (*F*_2,24_ = 18.811, *p* < 0.001), while PC2 separated aerial feeding events occurring at maximal prey heights from both aquatic feeding and aerial feeding at lower prey heights (*F*_2,24_ = 11.366, *p* < 0.001) (figure 3). Variables that loaded positively on PC1 primarily related to the timing of cranial events, with the lower scores exhibited during aerial feeding suggesting more rapid jaw movements (table 1). Time spent above 75% of peak velocity also loaded positively on PC1, while peak velocity, peak acceleration, peak gape size and maximal hyoid depression loaded negatively on this component. Strike distance and peak gape duration loaded positively on PC2, in addition to several timing-related variables (table 1). Premaxillary protrusion did not load strongly on either component.

**Table 1.** Principal component loadings for the aerial/aquatic PCA. Loadings with absolute values above 0.4 are bolded.

variable	PC1 (34.67%)	PC2 (25.53%)
peak velocity	**−0.784**	0.394
peak acceleration	**−0.758**	0.307
time above 75% of peak velocity	**0.536**	0.236
peak maxillary protrusion	0.216	−0.151
peak gape size	**−0.830**	0.073
peak hyoid depression	**−0.768**	0.151
strike distance	−0.355	**0.823**
time to peak gape	**0.510**	**0.743**
peak gape duration	−0.333	**0.739**
time to onset hyoid depression	**0.550**	**0.713**
time to onset cranial elevation	0.054	**0.848**
time to peak cranial elevation	**0.667**	0.018
time to peak hyoid depression	**0.460**	0.254
mouth closing duration	**0.751**	−0.205
strike duration	**0.614**	**0.603**

**Figure 4.**
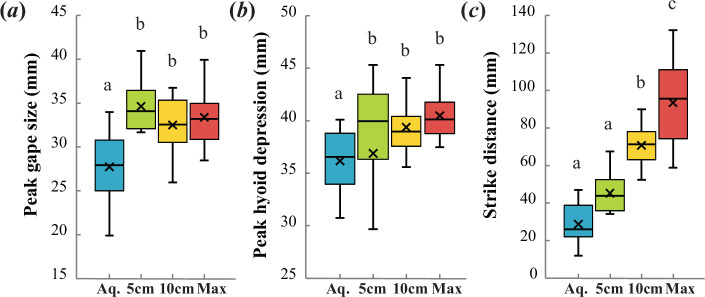
Graphs showing (*a*) peak gape size (*F*_3,21_ = 12.092, *p* < 0.001), (*b*) peak hyoid depression (*F*_3,21_ = 8.046, *p* < 0.001) and (*c*) strike distance (*F*_3,21_ = 42.004, *p* < 0.001) of aquatic (aq; *n* = 14) and aerial feeding events at prey heights of 5 cm (*n* = 14, except (*b*) where *n* = 13), 10 cm (*n* = 12), and individual maximal heights (*n* = 10). X indicates the mean, and whiskers show the range. Letters denote statistically significant differences at *α* = 0.05.

A series of repeated measures ANOVAs conducted on key kinematic variables further reinforced the differences between aerial and aquatic feeding. During aerial feeding events, largemouth bass displayed larger peak gapes (*F*_3,21_ = 12.092, *p* < 0.001) and greater hyoid depressions (*F*_3,21_ = 8.046, *p* < 0.001). In addition, aquatic strikes were generally initiated closer to the prey than aerial strikes (*F*_3,21_ = 42.004, *p* < 0.001), although the repeated measures ANOVA did not differentiate aquatic feeding and feeding on prey suspended at 5 cm above the surface in this respect (figure 4).

**Figure 5.**
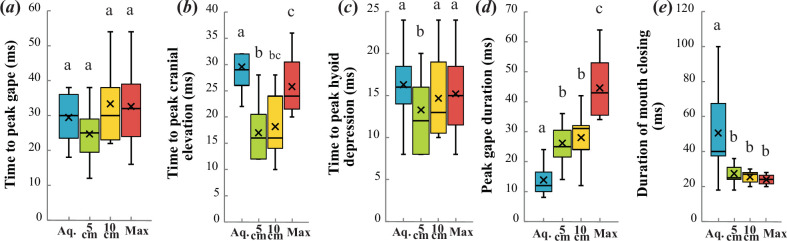
Graphs showing (*a*) time to peak gape (*F*_3,21_ = 1.987, *p* = 0.147), (*b*) time to peak cranial elevation (*F*_1.516,10.615_ = 11.882, *p* = 0.003), (*c*) time to peak hyoid depression (*F*_3,21_ = 4.182, *p* = 0.018), (*d*) peak gape duration (*F*_3,21_ = 38.327, *p* < 0.001) and (*e*) duration of mouth closing (*F*_1.371,9.594_ = 15.100, *p* = 0.002) of aquatic (aq; *n* = 14) and aerial feeding events at prey heights of 5 cm (*n* = 14), 10 cm (*n* = 12) and individual maximal heights (*n* = 10). X indicates the mean, and whiskers show the range. Letters denote statistically significant differences at *α* = 0.05.

There was also additional support for several differences in the duration of movements of the jaw and other cranial elements (figure 5). The time to peak cranial elevation (*F*_1.516,10.615_ = 11.882, *p* = 0.003) and duration of mouth closing (*F*_1.371,9.594_ = 15.100, *p* = 0.002) were both significantly shorter in aquatic versus aerial feeding events, while peak gape was sustained significantly longer during aerial prey capture (*F*_3,21_ = 38.327, *p* < 0.001). Time to peak hyoid depression was lower during aerial feeding at prey heights of 5 cm than during aquatic feeding (*F*_3,21_ = 4.182, *p* = 0.018), though there were no significant differences between any other categories. Repeated measures ANOVA found no significant differences in time to peak gape (*F*_3,21_ = 1.987, *p* = 0.147).

### (c) Differences between aerial feeding at varying prey heights

**Figure 6.**
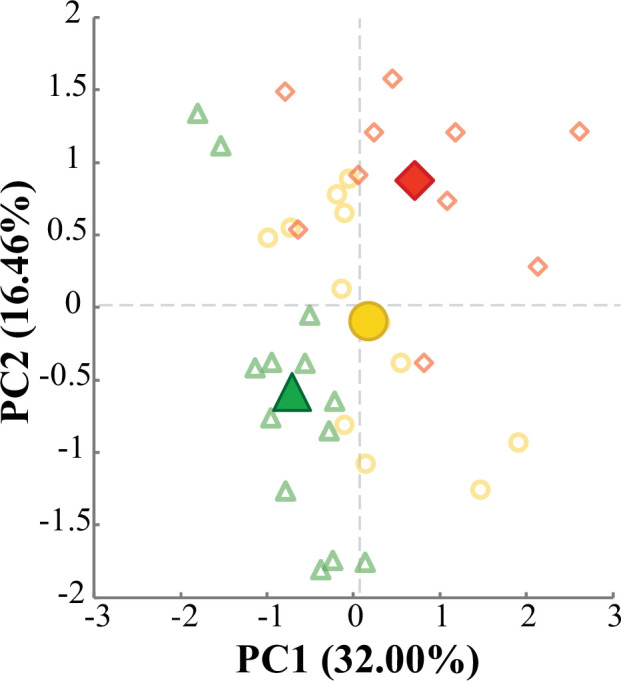
PC1 and PC2 scores of aerial feeding events at prey heights of 5 cm (open green triangles; *n* = 13), 10 cm (open yellow circles; *n* = 12) and at individual maximum prey heights (open red diamonds; *n* = 10). Filled shapes denote mean PC scores for each category. Time above 50% of peak velocity, number of propulsive tail strokes and time to initiation and completion of several jaw movements loaded positively on PC1, while attack depth loaded negatively. Peak velocity, peak acceleration, strike distance and peak gape duration loaded positively on PC2. Complete loadings are listed in table 2. Repeated measures ANOVA indicated significant differences along both PC1 (*F*_1.149,8.040_ = 8.432, *p* = 0.018) and PC2 (*F*_2,14_ = 10.120, *p* = 0.002).

The PCA performed using only aerial feeding data described the kinematic differences among the three prey height categories. PC1 and PC2 explained 48.46% of the total variance, and scores on both axes tended to be higher in each successive prey height category than the last (figure 6). Though scores were not significantly different between the 5 and 10 cm prey height categories, the maximal prey height category scored significantly higher than each of the others on PC1 (*F*_1.149,8.040_ = 8.432, *p* = 0.018) and PC2 (*F*_2,14_ = 10.120, *p* = 0.002). As the previous analysis indicated, variables that loaded positively on PC1 suggest jaw movements occurred more slowly as prey height increased. In addition, total strike duration, strike distance, number of propulsive tail strokes and time spent above 50% of peak velocity loaded positively on this axis, while peak gape size and attack depth loaded negatively (table 2). Peak velocity, peak acceleration, maximal hyoid depression, strike distance, number of propulsive fin strokes, peak gape duration and time to maximum cranial elevation loaded positively on PC2 of this analysis, while time to mouth closure loaded negatively (table 2). Overshoot loaded negatively on PC1 and positively on PC2.

**Table 2.** Principal component loadings for the aerial-only PCA. Loadings with absolute values above 0.4 are bolded.

variable	PC1 (32.00%)	PC2 (16.46%)
peak velocity	−0.363	**0**.**677**
peak acceleration	−0.232	**0.664**
time above 50% of peak velocity	**0.758**	0.050
peak maxillary protrusion	−0.018	0.061
peak gape size	**−0.501**	0.332
peak hyoid depression	−0.307	**0**.**628**
strike distance	**0**.**698**	**0**.**565**
pre-attack body angle	0.223	−0.032
jump angle	0.179	0.214
attack depth	**−0.466**	−0.373
propulsive fin cycles (#)	**0**.**612**	**0**.**469**
time to peak gape	**0**.**870**	−0.187
peak gape duration	**0**.**564**	**0**.**548**
time to onset hyoid depression	**0**.**897**	0.062
time to onset cranial elevation	**0**.**791**	−0.150
time to peak cranial elevation	0.353	**0**.**470**
time to peak hyoid depression	**0**.**547**	−0.262
mouth closing duration	0.098	**−0.551**
strike duration	**0**.**908**	0.061
overshoot	**−0.607**	**0**.**446**

**Figure 7.**
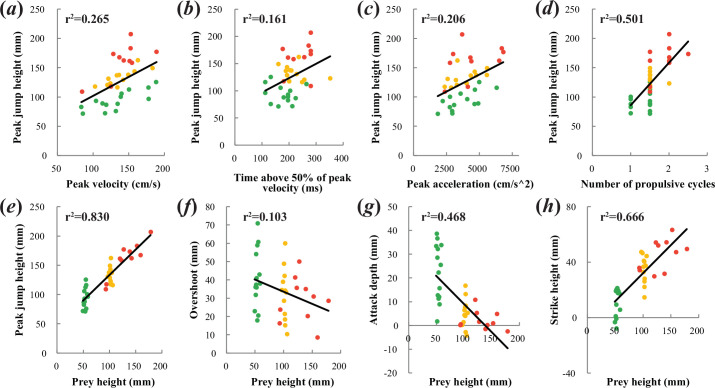
Top row: regressions of jump height against (*a*) peak velocity (*F*_1,34_ = 12.258, *p* = 0.001), (*b*) time above 50% of peak velocity (*F*_1,34_ = 6.514, *p* = 0.015), (*c*) peak acceleration (*F*_1,34_ = 8.836, *p* = 0.005) and (*d*) number of propulsive tail cycles (*F*_1,34_ = 34.136, *p* < 0.001). *n* = 36. Bottom row: regressions of prey height against (*e*) peak jump height (*F*_1,34_ = 166.35, *p* < 0.001), (*f*) overshoot (*F*_1,34_ = 3.916, *p* = 0.056), (*g*) attack depth (*F*_1,34_ = 29.899, *p* < 0.001) and (*h*) strike height (*F*_1,34_ = 67.919, p =< 0.001). *n* = 36.

Linear regressions found positive relationships between jump height and peak velocity (*F*_1,34_ = 12.258, *p* = 0.001), peak acceleration (*F*_1,34_ = 8.836, *p* = 0.005), duration spent above 50% of peak velocity (*F*_1,34_ = 6.514, *p* = 0.015) and the number of propulsive fin strokes used in a feeding event (*F*_1,34_ = 34.136, *p* < 0.001) (figure 7). Attack depth decreased as prey height increased (*F*_1,34_ = 29.899, *p* < 0.001), and there was a positive relationship between prey height and strike height (*F*_1,34_ = 67.919, *p* = < 0.001). Jump height also increased with prey height (*F*_1,34_ = 166.35, *p* < 0.001). There was a clear trend toward decreasing overshoot as prey height increased, though this relationship fell marginally short of significance at *α* = 0.05 (*F*_1,34_ = 3.916, *p* = 0.056) (figure 7). There was no effect of time elapsed between aerial and aquatic trials on aquatic time to peak gape (*F*_1,12_ = 0.000, *p* = 0.994), gape size (*F*_1,12_ = 0.698, *p* = 0.420) or peak velocity (*F*_1,12_ = 0.670, *p* = 0.429).

Pairwise comparisons following repeated measures ANOVA found that maximum gape size, peak hyoid depression, time to peak gape, time to maximal hyoid depression and time to mouth closure were not different across any aerial prey height category (figures 4 and 5). Strike distance increased with prey height, and each aerial height category was significantly different from the others (figure 4). Peak gape duration was not significantly different between feeding events at prey heights of 5 and 10 cm but was significantly longer during maximal feeding efforts. Lastly, time to peak cranial elevation was significantly greater at maximal prey heights as compared with 5 cm, but each of these prey height categories was not significantly different from the intermediate 10 cm prey height category in this respect (figure 5).

## 4. Discussion

Due to the inability of fish to suction feed during emersion, largemouth bass shifted their prey capture strategy when feeding aerially to instead overtake and engulf prey with large gapes. In addition, aerial feeding freed bass of the constraints of suction feeding and allowed them to initiate strikes far from the prey and maintain peak gape for long durations, thus maximizing the volume engulfed and possibly reducing the need for accuracy.

### (a) Aerial feeding in largemouth bass and other fishes

The aerial feeding behaviour of the largemouth bass shares a number of similarities with other species of fishes. First, they display the same general pattern of hovering, thrust production and gliding as archerfish, as described by [20]. Second, like the silver arowana, bass use S-starts to generate the thrust required to jump [14]. Many fishes, including largemouth bass [46] and especially those with elongate body shapes, use S-starts to ambush aquatic prey as they do not require any change in direction or body orientation [47–49]. The kokanee salmon (*Oncorhynchus nerka*) [34] and mangrove rivulus (*Kryptolebias marmoratus*) [13,50] also launch jumps out of the water using S-starts and, although there are no published records of these species feeding aerially, species closely related to each of these can capture aerial prey [15,16]. If S-starts are used during aerial feeding across such diverse groups as salmonids, rivulids, centrarchids and osteoglossids, it is possible that this strategy for generating thrust during aerial prey capture can be generalized to other taxonomic groups with similar body shapes.

### (b) Differences between aerial and aquatic prey capture

Our first prediction, that aerial feeding would be characterized by larger and faster movements of cranial structures involved in feeding, was supported (figures 3–5, table 1). Peak gape size and maximal hyoid depression were greater during aerial feeding, and times to peak cranial elevation and mouth closure were shorter (figures 4 and 5). The aquatic feeding kinematics reported here are comparable to those published in previous studies involving fish of similar size (electronic supplementary material, table S1), which suggests that they are representative of the species and resulted from maximal feeding efforts.

There are two possible explanations for the differences between aerial and aquatic feeding. First, largemouth bass may alter their prey capture strategy when feeding aerially to increase capture success. If so, the large gape size and long duration of peak gape during aerial feeding events could increase the volume engulfed by the mouth, thus reducing the targeting accuracy necessary to capture prey [14]. The ability of *M. salmoides* to correct for refraction is unknown, and minimizing the importance of aim may be important if this species lacks the ability to adjust for this distortion. Under this hypothesis, the rapid mouth closure that occurs during aerial feeding could be seen as a way of quickly trapping flying prey items within the jaws before they can escape in an environment where relying on water currents to capture and transport prey is impossible [21].

Alternatively, the differences in the extent and speed of cranial movements could stem from differences in the physical properties of air and water. Water is roughly 800 times denser and 50 times more viscous than air [12,51–53]. Thus, objects (and in this case, anatomical structures) moving through air experience only a small fraction of the drag that they would in water at any given velocity [52,53]. Consequently, it is possible that aerial jaw movements result from identical muscle activation but proceed farther and faster in air because they encounter less resistance due to drag. Electromyographic recordings of feeding and locomotor muscles during aerial and aquatic feeding would provide insight into this possibility.

One line of evidence that suggests at least some kinematic differences between aerial and aquatic feeding occur due to modulation of behaviour, rather than incidentally due to physical parameters, is the dramatic difference in strike distance that characterizes aerial and aquatic feeding events (figure 4*c*). During aquatic suction feeding, the mouth of the predator must be positioned close to the prey at strike initiation due to the rapid decay in flow speed that occurs as the predator–prey distance increases [8,37,54]. With this constraint removed in air, along with any added energetic cost due to drag that would be incurred by swimming a long distance with an open mouth under water, there may be a net benefit to opening the mouth early during aerial feeding to create a long peak gape duration, thereby maximizing the volume engulfed (as mentioned previously) [14]. If there is a net benefit to a long peak gape, this could explain the positive association between strike distance and prey height.

As predicted, peak velocities and accelerations were greater during aerial as compared with aquatic feeding events. The velocities follow the trend observed by Lowry *et al.* [14] in *O. bichirrosum*, which is likely necessary for overcoming gravity to reach prey far above the surface where fish are no longer buoyant. High peak accelerations are also likely necessary when fish must reach high velocities quickly given their relatively shallow position below the surface. The rapid vertical accelerations produced by the S-start coupled with the rapid decelerations due to gravity cause largemouth bass executing aerial feeding events to spend less time above 75% of their peak velocity. This was visually represented by steeper, more narrow velocity over time curves (electronic supplementary material, figure S2).

### (c) Aerial feeding at different heights

As expected, jump height increased with prey height (figure 7*e*). Additionally, overshoot tended to decrease as prey height increased, though not significantly (figure 7*f*). Shih *et al.* [20] found a weak positive relationship between prey height and overshoot in *Toxotes*, although these data suffered from conflicting trends within individuals and limited representation of individuals across a range of prey heights. Nevertheless, it is possible that largemouth bass can less accurately judge prey height than archerfish, leading them to overshoot when the prey’s location is uncertain and performance allows, as overshooting is more likely to result in prey capture than undershooting [20]. If so, the trend towards decreasing overshoot at high prey heights could simply have resulted from individuals nearing their maximal jump performance, forcing them to overshoot the prey by smaller margins.

In accordance with our predictions, largemouth bass reached greater heights by accelerating more rapidly and generating higher peak velocities (figure 7). They also modulated their duration spent travelling at high velocities (figure 7*b*). This is likely possible because bass rarely leave the water completely and can therefore continue generating thrust when most of the body has emerged, if necessary. Displacement is a function of both velocity and time, and in the case of aerial feeding, bass appear to increase both to achieve sufficient heights. Jump angle and body angle at attack initiation do not appear to change with prey or jump height.

High velocities and long durations above 50% of peak velocity, both factors that contribute to jump height, are in turn achieved (though probably not exclusively) by increasing the number of propulsive tail stroke cycles. Shih *et al.* [20] found similar trends in archerfish, but also noted that the amplitude and frequency of each stroke both vary widely and likely contribute to thrust generation. Amplitude and frequency were not measured in the present study and could further explain variation in jumping performance.

Largemouth bass initiated jumps closer to the surface at greater prey heights (figure 7*g*). We predicted that deeper attack initiations would be associated with higher jumps, as this would give the fish greater distances over which to accelerate to higher velocities. Migrating salmonids clear higher obstacles when given adequate pool depths, apparently for this reason [33–35], and Trinidadian guppies (*P. reticulata*) likewise jump higher after increasing their available acceleration distance by swimming backwards farther before jumping [36]. The fishes analysed in these studies, however, were not jumping to capture prey and did not require high accuracy. Aerially feeding largemouth bass, in contrast, must see a small target across some distance spanning water and air. Even completely pure water, which is virtually non-existent in natural systems, attenuates light, and it may be advantageous for bass to minimize the distance light must travel through water between their eyes and the prey, especially when the prey is distant [52,55]. In addition, minimizing the distance between the predator’s eye and a distant prey increases the visual angle occupied by the prey item, making it appear larger in the field of vision of the predator [56].

If there are meaningful visual advantages to initiating attacks near the surface, one question remains: why do bass initiate attacks from deeper in the water column when capturing prey suspended at lower heights? The same fish are capable of capturing prey that is suspended much higher when attacking from just below the surface, so any added velocity generated by increasing the acceleration distance is likely not necessary. First, qualitative observations of aerial feeding events show that the bass often briefly rose to the surface shortly before initiating attacks at low heights. This may provide the fish with all visual information necessary to determine the location of the prey. Next, we speculate that the bass return to greater depths before mounting attacks to increase the time elapsed between attack initiation and prey capture. This helps ensure that key jaw movements, such as reaching peak gape, are executed before the prey is approached, which may increase capture success. Our results suggest that key jaw movements onset earlier and occur more rapidly at lower prey heights (figure 6), which lends support to this hypothesis. Finally, it is possible that bass initiate strikes deeper in the water column when prey is near the surface in order to avoid prematurely startling the prey.

## 5. Conclusions and future directions

Many of the fundamental kinematics underlying aerial feeding in largemouth bass are shared with the two previously studied species. Like the silver arowana, *O. bichirrosum*, bass generate the acceleration necessary to leap above the surface by performing S-starts. Similarities between these two species extend further, with aerial feeding in both generally involving larger and more rapid movements of the jaw apparatus as compared with aquatic prey capture [14]. Like archerfish (*T. microlepis*), largemouth bass jumped higher as prey height increased by generating higher peak velocities and greater peak accelerations [20]. They also spent more time near peak velocity at greater prey heights, and both species generated higher jumps by increasing the number of propulsive tail beat cycles during the approach.

To our knowledge, this study is the first to explore aerial feeding in a species with a largely generalized teleost body shape, mouth morphology and aquatic ram/suction feeding behaviour. As the aerial prey capture behaviours of more species are described, it may become possible to begin identifying shared morphological traits associated with this ability. For example, of the three species (*O. bicirrhosum*, *T. microlepis* and *M. salmoides*) in which aerial prey capture has been examined, two (*O. bicirrhosum* and *M. salmoides*) have large mouths that may help engulf prey [14]. These species also have moderately elongate bodies and relatively large body and fin areas, especially caudally, which are characteristics associated with species capable of rapid acceleration and S-starts [57,58]. While the last (*T. microlepis*) differs in its overall body form, it also has a large lateral caudal surface area. It is possible that these (and other, yet to be identified) characteristics can make a fish pre-adapted for aerial feeding, creating the circumstances for exaptation to occur.

Still, little is known about aerial feeding by fishes. Future work should investigate whether kinematic differences between aerial and aquatic feeding result incidentally due to differences in the physical properties of air and water or result from changes in muscle activation. It is also unknown whether the ‘S-start’ displayed by several species during aerial feeding mirrors muscle activation patterns of true aquatic S-starts. Studying aerial feeding across closely related taxa that vary in their attack strategies would also shed light on their ability to modulate kinematics. Largemouth bass are known as generalists, displaying fairly flexible feeding behaviours [59]. Therefore, comparing this species of centrarchid to one that feeds in a relatively stereotyped way, such as the green sunfish (*Lepomis cyanellus*) [59], would provide important information regarding the behavioural constraints on feeding aerially. Lastly, the ecological role of aerial feeding by non-specialists such as the largemouth bass warrants attention. For example, the ability to exploit aerial prey could give fishes capable of aerial feeding a competitive advantage against other species, especially when aquatic prey are scarce, or simply reduce the predation pressure on aquatic prey regardless of their abundance. Both of these scenarios could have far-reaching implications for ecosystem dynamics.

## Data Availability

Data are available in the electronic supplementary material [60].
